# Supplementary material for: Acceptance and compliance with micronutrient powder (MNP) among children aged 6–23 months in northern Nigeria
Source: PLOS Glob Public Health. 2022 Oct 17;2(10):e0000961. doi: 10.1371/journal.pgph.0000961 (PMC10022258; doi:10.1371/journal.pgph.0000961)
Supplement: S1 Dataset — (ZIP) [file pgph.0000961.s008.zip › S8_Dataset/Spot Checks.zip!/Spot Checks/06072015_A_GA_AS_2_SC a.pdf]

SPOT CHECK RECORDING FORM

State: Adamawa **LGA: ____Ganye___ Community: _Sandoka_**

Name of caregiver (female): Hafsatu Haruna

Name or caregiver (male): ………………………

Sex of index child: _Male___ **Age of index child (in months): _9 months_______**

Spot Check Information

1. **Date of MNP distribution to household** (circle option below based on LGA name above)**:**
   - **Kebbi:** BirninKebbi&Jega LGA: **June 16**
   - **Kebbi:** Danko-wasagu: **June 17**
   - **Adamawa:**Song, Ganye: **(June 18)**
   - **Adamawa:**Guyuk **June 19**
2. **Date of Spot Check Observation: ____6/7/2015_____**
3. **Number of sachets remaining**(Ask the caregiver to show you and count the specific number of sachets remaining)**:**

   41

4. **Number of sachets that <u>should remain</u>using one MNP sachet per day** (Analyst only)**:**

   43

5. **Number of sachets remaining / number of sachets should be remaining**(Analyst only)**:**

   _____41____ / ___43______ = _95.3___ %

Probes

Question 1. You have **XX** MNP sachets remaining. Explain how easy or difficult it was to use the MNP product on a daily basis for your child 6 – 23 months? (Circle one answer)

Very Difficult **(Difficult)** **Easy** **Very Easy**

Question 2. Describe any specific challenges that you faced that made the MNP difficult to use on a daily basis?
The sachet of the MNP is to thick and therefore makes it difficult for me to use or to unseal and also the MNP takes like 2 to 3 minutes to dissolve into kunu. These are my experience.

Question 3. Describe specific aspects of the MNP product that you or your child enjoyed that made it easier to use on a daily basis?

- After mixing the MNP into the kunu and I use a small spoon to feed my child. That way, it makes it easier for the child to accept the food. The fact that I use a small spoon daily to feed him is very easy for me.

Question 4. What questions do you have about the MNP based on your experience using it?

- Due to current health condition of my child (he is suffering from the attack of evil spirit). We use order herbs and leaves other means like prayers for the child. I want to know if I should continue to administer the MNP and still use those herbs and leaves for the child because of his present condition.

Question 5. What specific recommendations do you have that we can do as a program to make the MNP easier to use on a daily basis?

The MNP small sachet should be made in a paper form or a paper sachet so that it would be made easy for us to use on a daily basis because sometimes I use razor blade to tear the sachet and we all know as a mother you are not expected to keep razor blade anywhere close because of other children that is only recommendation regarding this product.
